# Emotional Regulation Challenges in Chilean Teachers: An Analysis of the Measurement Invariance of the DERS-E and the Influence of Gender and Age

**DOI:** 10.3390/jintelligence12090086

**Published:** 2024-09-03

**Authors:** Flavio Muñoz-Troncoso, Enrique Riquelme-Mella, Amy G. Halberstadt, Ignacio Montero, Valeria Sepúlveda-Bernales, Gerardo Fuentes-Vilugrón, Edgardo Miranda-Zapata, Ekaterina Legaz-Vladímisrkaya, Felipe Caamaño-Navarrete, Gerardo Muñoz-Troncoso

**Affiliations:** 1 Faculty of Education, Universidad Católica de Temuco, Temuco 4810296, Chile; 2 Faculty of Social Sciences and Arts, Universidad Mayor, Temuco 4801043, Chile; 3 Department of Psychology, North Carolina State University, Raleigh, NC 27606, USA; 4 Faculty of Psychology, Universidad Autónoma de Madrid, 28049 Madrid, Spain; 5 Faculty of Education, Universidad Autónoma de Chile, Temuco 4810101, Chile; 6 Collaborative Research Group for School Development (GICDE), Temuco 4810101, Chile; 7 Faculty of Social Sciences and Humanities, Universidad Autónoma de Chile, Santiago 7580150, Chile; 8 Faculty of Philosophy and Humanities, Universidad Austral de Chile, Valdivia 5110566, Chile; 9 Faculty of Education, Universidad San Sebastián, Valdivia 5110693, Chile

**Keywords:** emotional dysregulation, Chilean teachers, measurement invariance, gender and age, emotional training, teacher well-being

## Abstract

The study investigates the emotional dysregulation in teachers of the Chilean school system, focusing on gender and age similarities and differences. The sample included 1059 teachers from various regions of Chile, of whom 80.3% were female and 19.7% were male. Participants completed the Spanish version of the Difficulties in Emotional Regulation Scale (DERS-E). A confirmatory factor analysis was carried out to evaluate the structure of the theoretical model, along with the convergent, discriminant, and internal consistency of the instrument. Additionally, a measurement invariance analysis was performed to identify possible differences between demographic groups, which is crucial to ensure that comparisons between these groups are valid and unbiased. The results indicated that the theoretical model presents a good fit to the data and confirms the validity and reliability of the DERS-E. Scalar invariance was achieved among the analyzed groups. We found significant differences in emotional dysregulation between men and women, which also varied by teacher age. The importance of understanding the specific needs of teachers in terms of their emotional regulation is discussed and the urgency of implementing training programs that improve their emotional skills, fostering a positive and effective learning environment, is highlighted.

## 1. Introduction

Emotional regulation (ER) is a process that allows individuals to transform, minimize, or intensify emotions depending on the context and the objective to be achieved (Gross and Thompson 2007; Thompson 2014). This implies that people have the ability to manage emotions to a certain level, estimating their consequences and acting in a particular way (Hewitt Ramírez et al. 2022), which makes it possible to maintain that it is a skill. Although many people do not think they can manage their experience of emotions as well as their expression of emotion, researchers have argued effectively that we can indeed manage our internal experiences (Ford et al. 2018; Ford and Gross 2018; Olhaberry and Sieverson 2022; Salguero and Ramos-Cejudo 2023). Further, the ability to regulate emotions has been understood as a central component of affective competence that is possible to develop and utilize throughout one’s lifespan (Camras and Halberstadt 2017; Halberstadt et al. 2001; Isaacowitz and English 2024; Morris et al. 2017).

Emotional regulation (ER) is a vital skill for teachers, impacting their well-being and the effectiveness of their teaching. The ability to manage emotions not only affects teachers’ personal and professional lives but also plays a crucial role in shaping students’ learning experiences (Schonert-Reichl 2017; Taxer and Gross 2018). Studies have shown that teachers with strong emotional regulation skills are better equipped to handle classroom challenges, leading to improved academic performance and student–teacher relationships (Brackett et al. 2010; Castillo-Gualda et al. 2019). The importance of emotional regulation is further emphasized by its connection to psychological well-being and mental health, highlighting the need for targeted interventions to support teachers in developing these skills (Extremera et al. 2019; Guzmán-González et al. 2014).

How we regulate emotions also has important implications. Indeed, the type of strategies one chooses in achieving emotional regulation (ER) is now seen as a key to general well-being as well as to building healthy bonds with others (Ford et al. 2018; Olhaberry and Sieverson 2022). It is worth mentioning mindfulness and reappraisal as adaptive strategies (Navas-Casado et al. 2023), which are thought to result in greater success for specific regulatory goals and better long-term physical and mental health outcomes (Taxer and Gross 2018; McRae and Gross 2020).

ER skills are acquired over the lifespan of an individual, with increasing capacity during development. At the same time, while we have increasing capacity, with increases in executive function and awareness of cultural norms, individual experiences and the understanding of one’s own life stories guide our use of various strategies. In turn, the applied strategies can be diverse and used simultaneously in the same situation (Taxer and Gross 2018). Thus, the choice of strategies, their effectiveness, and the way in which they are used for ER are individual and multidimensional, and develop over time.

Although parents are usually considered the primary socializers of emotion regulatory strategies (Morris et al. 2017), children acquire many ER values, beliefs, and strategies from others, including siblings, peers, and educators (Ceballos-Marón et al. 2022). School is a space where a considerable amount of emotion, socialization, and transmission of knowledge occurs (Taxer and Gross 2018; Fuentes-Vilugrón et al. 2022; Muñoz-Troncoso et al. 2024). There is increasing evidence about the importance of ER in educational processes, especially given the amount of time that children spend in school settings and the accepted social and educational power wielded by teachers. Indeed, the burgeoning Socio-emotional Learning (SEL) movement recognizes the ability of teachers to socialize children’s emotion understanding, expression, and experience (Schonert-Reichl 2017).

Given the powerful role of teachers in guiding children’s socio-emotional competence, it is critical to better understand teachers’ own abilities toward emotion regulation, as an essential component in the interaction of the teacher with the student and, therefore, in educational processes (Rodriguez and Kross 2023). Despite the importance of teachers in the school setting and in the role of guiding students in socioemotional ways (De la Cueva and Montero 2018; Montero and De la Cueva 2020), efforts to study teachers’ emotion regulation, and, indeed, their dysregulation, are only growing now (Taxer and Gross 2018; Legette et al. 2021).

### 1.1. Emotional Dysregulation

Linehan’s (1993) definition of Emotional Dysregulation (ED) is the most accepted among cognitive behavioral therapies and behavioral interventions. This author conceptualized ED as ‘a high emotional vulnerability to regulate emotion [...] as well as a deficit in the ability to modulate emotions’ (Linehan 1993). Although this definition emphasizes the morphological characteristics of emotionally dysregulated responses, it ignores those factors that alter emotional behaviors (Cole et al. 1994; Gratz 2007). Likewise, such a definition does not explain ER, which is the center of ED from that perspective.

When the biological and learning components of ER are configured in a non-adaptive manner, they result in patterns of dysregulated behavior that can give rise to psychological disorders such as anxiety, depression, borderline personality disorder, substance use, and alterations in eating behavior (Paredes-Rivera et al. 2021; Preece et al. 2021; Vizioli 2022). It should be noted that, in addition to psychological difficulties, ED is associated with other problems that can alter well-being or functioning in the physical dimension, such as hypertension, cancer, and self-harm behaviors (Ciuluvica et al. 2020). Likewise, in the social dimension, there may be a lack of empathy or coexistence problems (Fuentes-Vilugrón et al. 2022).

### 1.2. Teacher Emotional Regulation

The decision to employ the DERS in a teacher population stems from the unique emotional challenges faced by educators that may not be fully captured by tools designed for the general population. Teachers frequently navigate complex emotional landscapes involving high-stress interactions with students, colleagues, and parents, often under significant time constraints. These stressors may amplify emotional dysregulation in ways distinct from other professions, necessitating a specialized tool like the DERS that can account for these profession-specific emotional dynamics (Taxer and Gross 2018; Brackett et al. 2010). Furthermore, the DERS’s validation in varied cultural settings, including Chile, provides a strong basis for its use in this specific educational context, ensuring that the tool accurately reflects the emotional experiences of teachers and facilitates the development of targeted interventions to improve their emotional well-being (Guzmán-González et al. 2014).

Emotional dysregulation is often rooted in past traumatic experiences, and teachers, as a professional group, may be particularly vulnerable to such histories. Research indicates that adverse childhood experiences (ACEs) are prevalent among educators, potentially contributing to heightened emotional dysregulation in stressful educational environments (Felitti et al. 1998; Anda et al. 2006). Although teachers’ own experience of childhood emotional abuse is associated with sensitivity to children’s own experiences in school and thus the greater identification and reporting of these cases (Fabris et al. 2024), it is likely to increase teachers’ own reliving of such experiences and thus their personal stress while teaching. This connection underscores the need for specialized tools like the DERS, which can sensitively capture the nuanced ways in which past trauma continues to influence emotional regulation in the present, particularly in the high-stress context of teaching (Briere and Scott 2015).

The ER capacity of teachers is thought to facilitate both the development of ER in students and a classroom context which facilitates learning (Borja-Sarmiento 2022; Ordoñez Rodríguez 2022). Teachers play a critical role in the emotional and psychological development of students, functioning as ad hoc attachment figures within the school environment. As attachment figures, teachers contribute significantly to students’ emotional socialization, influencing how students perceive, manage, and express their emotions (Spilt and Koomen 2022; Longobardi et al. 2019). Emotionally regulated teachers also provide an important security base from which students can explore both academic and social terrain in and outside of the classroom, improving students’ levels of well-being, and reducing the frequency of disruptive behaviors (Hewitt Ramírez et al. 2022). Likewise, ER impacts other situations that can influence student trajectories such as coping with risk situations, preparing for exams, using technology, and coping with stressful life situations (Santander-Trigo et al. 2020).

In relation to the well-being of teachers, ER has a determining role. First, it helps teachers to improve their own effectiveness, with the understanding that it is necessary to manage a certain amount of emotion in the classroom (Taxer and Gross 2018). Teachers’ ER has been associated with the importance they give to specific training in emotional regulation, the amount of resources allocated for this, and motivation in the face of students’ lack of ER (Santander-Trigo et al. 2020). Second, ER is relevant for managing stress and emotional exhaustion, typical for an emotionally challenging job such as the teaching profession (Donker et al. 2020), which involves adjusting to the requirements of the classroom, the organizational culture, and the expectations of students and parents. As such, developing and maintaining ER strategies is a permanent challenge for educators (Castillo-Gualda et al. 2019).

There is broad consensus that the ER of subjects is capable of influencing their well-being, as well as being relevant for the development or maintenance of psychological disorders (Sánchez-Gómez et al. 2022). In this sense, there is evidence regarding its role in educational processes and the importance of ER in teachers. Likewise, there is an increase in interest in the study of ER for the management of training models and the actions that can be carried out in relation to emotionally regulated teachers (Fuentes-Vilugrón et al. 2022).

Teachers’ emotional dysregulation is closely tied to the frequent and intense negative emotions experienced at the workplace, often triggered by factors such as classroom management challenges, a lack of administrative support, and high-stakes testing environments (Taxer and Gross 2018). Additionally, the emotional demands of responding to students’ behavioral issues and maintaining parental relationships under stressful conditions can further exacerbate negative emotional states. These stressors are not only frequent but also multifaceted, affecting teachers’ ability to regulate emotions effectively and contributing to burnout and decreased job satisfaction (Brackett et al. 2010).

One implication of ER is the modulation of the expression and tone of emotion. Firstly, where there is support from adults, the child can develop more independent strategies such as distraction and distancing (Vindel and Montero 2010). They can redefine these strategies and use others for specific contexts and people, such as imagination and fantasy or cognitive problem-solving strategies (Denham et al. 2007).

### 1.3. Instruments to Evaluate Emotional Regulation

At the international level, a wide variety of instruments allow ER to be evaluated at different stages of development, from childhood to adulthood, with multiple forms of evaluation provided for adults, including questionnaires, implicit association measures, and interviews (Table 1). The goals also vary from assessing one’s orientation to and value for emotion experience (e.g., Katz and Gottman 1999; Hakim-Larson et al. 2006) to reporting on the use of specific emotion regulatory strategies (Gross and John 2003; Rovira et al. 2012) to focusing on specific types of dysregulation such as anger (Hoshmand and Austin 1987). From the existing measures, we chose a questionnaire design as the most practical for assessing a large sample of adults, and assessing the generalizability of structure across gender, age, and regionality. We particularly wanted a measure that focused on dysregulation as we considered it important to first identify the issues that individuals would need to address to choose the emotion regulatory skills to develop. That is, individuals who lack self-awareness and understanding about emotion might need to develop different strategies, such as emotion recognition skills or attention to somatic cues whereas others who become highly reactive when distressed might need to alter catastrophic thinking patterns or work on breathing techniques to reduce their own distress. Thus, we turned to the DERS, with its comprehensive measurement of the different components of experience associated with dysregulation.

### 1.4. The DERS

The decision to use the DERS over other instruments listed in Table 1 was driven by its comprehensive assessment of emotional dysregulation, which is central to the study’s objectives. Unlike other tools, the DERS provides a multidimensional evaluation of emotional regulation difficulties, encompassing aspects such as emotional clarity, impulse control, and goal-directed behavior, which are crucial for understanding the emotional challenges faced by teachers. Additionally, the DERS has been validated in various cultural contexts, including in Chile, ensuring its relevance and applicability to the studied population (Guzmán-González et al. 2014; Hervás and Jódar 2008).

The difficulties in emotional regulation scale (DERS) (Gratz and Roemer 2004) in its original form included six factors: (1) non-acceptance of emotional responses; (2) difficulties in goal-directed behaviors when upset; (3) difficulties controlling impulsive behaviors when upset; (4) limited access to emotional regulation strategies perceived as effective; (5) lack of emotional awareness; and (6) lack of emotional clarity. Hervás and Jódar (Hervás and Jódar 2008) recommend merging the factors ‘difficulties in controlling impulsive behaviors when upset’ and ‘limited access to emotional regulation strategies perceived as effective’, into one called ‘emotional lack of control’. This gives rise to the DERS-E, composed of five factors: (1) emotional rejection; (2) everyday interference; (3) emotional neglect; (4) emotional lack of control; and (5) emotional confusion.

The DERS has been widely cited and studies have explored the convergent and discriminant validity of the DERS in different populations and contexts, confirming its usefulness in measuring emotional dysregulation (Gross and John 2003; Garnefski et al. 2001; Gratz and Roemer 2004). Some validations have been carried out in South America, including with a university population in Argentina (Medrano and Trógolo 2014), and Colombia (Muñoz-Martínez et al. 2016). In Chile, a validation of the version adapted to Spanish (DERS-E) was carried out in the general population (Guzmán-González et al. 2014). Research in Chile on the instrument has analyzed the psychometric properties of the DERS-E version by Hervás and Jódar (Hervás and Jódar 2008), considering its reliability and validity of both construction and criteria. These studies highlight the importance of validating and adapting the scale to specific cultural contexts to ensure the accuracy and relevance of its results (Hervás and Jódar 2008; Medrano and Trógolo 2014, 2014; Muñoz-Martínez et al. 2016). This study builds on the existing foundation in three ways.

First, we expand on knowledge regarding dysregulation by age and gender. The choice to assess invariance by age and gender is based on previous studies that have demonstrated significant differences in emotional regulation as a function of these factors (Gross and John 2003; Gratz and Roemer 2004). Measurement invariance in these dimensions is crucial to ensure that any observed differences in emotional regulation are not the product of inherent biases in the scale itself, but of true differences in emotional experiences between these groups (Guzmán-González et al. 2014; Muñoz-Martínez et al. 2016).

Second, we expand our understanding of dysregulation levels to Chilean teachers who may or may not have different experiences than university students or the general populace. The assessment of the dysregulatory aspects of the emotion experience of teachers is important; this is because, as noted above, emotionally dysregulated teachers are less effective in being able to cope with classroom challenges, particularly when they are needed to resolve emotionally laden situations with students.

Third, within the population of teachers, we also included educational level as a category of analysis because emotional demands and regulation strategies may vary significantly between primary and secondary education levels. Teachers at different educational levels may face different types of stress and emotional challenges, which could influence their capacity for emotional regulation (Borja-Sarmiento 2022; Ordoñez Rodríguez 2022). Therefore, it is crucial to determine whether the DERS-E scale consistently measures emotional dysregulation across these different developmental and educational contexts.

### 1.5. The Current Study

Given the aforementioned context, the objectives of the present study are (1) to evaluate emotional dysregulation among teachers in the Chilean school system, differentiating by gender and age groups to understand the specific emotional challenges within this population, (2) to determine the measurement invariance of the Difficulties in Emotion Regulation Scale (DERS-E) across different teacher groups, such as the Gender, Macrozone, and Educational levels, ensuring that the scale consistently measures emotional dysregulation across these groups and allows for valid comparisons, and (3) to explore how emotional dysregulation impacts teachers’ ability to manage classrooms and engage with students, contributing to the development of targeted interventions that enhance teachers’ emotional well-being and the overall quality of the educational environment.

The decision to use the DERS in the teacher population, rather than relying on a version adapted for the general population, is rooted in the unique emotional demands placed on educators. Teachers encounter specific stressors in their professional environment, such as managing classroom behavior, interacting with students of diverse backgrounds, and coping with administrative pressures, all of which can exacerbate emotional dysregulation (Taxer and Gross 2018; Brackett et al. 2010). The DERS, with its comprehensive assessment of emotional regulation difficulties, offers a robust framework to explore these challenges in depth. Unlike general population tools, the DERS provides detailed insights into how emotional clarity, impulse control, and goal-directed behavior are specifically affected by the teaching context (Gross and John 2003; Gratz and Roemer 2004). Furthermore, the DERS has been validated in various cultural settings, including Chile, ensuring its relevance and applicability to the studied population (Guzmán-González et al. 2014). This study builds on these validations to assess how the instrument performs within the specific emotional landscape of educators, contributing to targeted interventions that enhance teacher well-being and effectiveness.

In view of the above, this research aims to evaluate the potential differences in emotional dysregulation between the groups that make up the teaching staff that participated in the study, for which the following hypotheses are proposed:

**H1.**  *There are statistically significant differences in emotional dysregulation scores between men and women. The above, based on the premise that gender socialization influences the way emotion is experienced and regulated, is suggested by many previous studies (Gross and John 2003; Gratz and Roemer 2004; Brody and Hall 2010).*

**H2.**  *Emotional dysregulation scores present variations in relation to age. A growing corpus of work indicates heterogeneity in the intensity of feeling across age, as well as improved emotional regulation strategies with experience and maturity (Guzmán-González et al. 2014; Muñoz-Martínez et al. 2016; DiGirolamo et al. 2023).*

**H3.**  *The variation in scores according to age is different between men and women. Based on previous research, we predicted that men and women would differ in their levels of emotion dysregulation, and that dysregulation might be attenuated with age. What is not yet well-studied is whether the gender differences exist across age, and whether the trajectory of the development of emotional regulation strategies is similar or different throughout life as a function of gender (Hervás and Jódar 2008; Medrano and Trógolo 2014).*

## 2. Materials and Method

The research is framed in a psychology research methodology, of a transversal quantitative type, with a comparative descriptive design, as stated by León and Montero (2015).

### 2.1. Participants

The sample was obtained by convenience according to accessibility criteria, with a total of 1059 participants, women (80.3%) and men (19.7%) with ages from 22 to 71 years (M = 37.89; SD = 9.22) and a length of service from 1 to 50 years (M = 11.41; SD = 8.41). The participants are teachers from the Chilean school system who teach in primary (60.7%) and secondary education (39.3%), in establishments in the North (12.47%), Center (31.35%), South (20.96%) and Metropolitan (35.22%) macrozones. It should be noted that, while the questionnaire offered options for other genders besides Female and Male, participants only selected these two options. This result could be due to the specific cultural and social context of the Chilean school system, which may influence self-identification with traditional gender categories. The inclusion criterion was being a teacher within the Chilean school system, excluding individuals who identified as being of a higher technical education or university faculty.

### 2.2. Instrument

The Difficulties in Emotion Regulation Scale (DERS) by Gratz and Roemer (2004) was applied, having been adapted to Spanish by Hervás and Jódar (2008) (DERS-E). The instrument has been validated in the Chilean population (2014) and a subsequent study that proposes reference values for it (2014). In addition, it was used in a recent investigation regarding the emotional self-regulation of Chilean teachers during the COVID-19 pandemic (Fuentes-Vilugrón et al. 2022). In the aforementioned studies, the results showed good reliability indices measured through Cronbach’s Alpha coefficient (from 0.71 to 0.92 for the subscales); however, it is unknown whether in Chilean teachers, the structure of the instrument has the same meaning for the groups it is proposed to explore.

The DERS-E is a 5-point Likert-type self-report scale, whose response options relate to the frequency that subjects attribute to the description of the indicators. Thus, the higher the score, the greater the difficulty of emotional regulation (1 = Almost never; 5 = Almost always). It has 25 items grouped into 5 latent variables: (1) Emotional rejection [ER], seven items about negative reaction to the emotional responses of oneself and others, e.g., ‘I feel ashamed when I have emotions’; (2) Emotional dyscontrol [ECD], six items about problems controlling one’s behavior when experiencing an emotion with high intensity, e.g., ‘When I’m upset, I have difficulty controlling my behaviors’; (3) Everyday interference [IC], four items about how emotions interfere with effective action toward a goal when people experience a negative emotion, e.g., ‘When I experience a negative emotion, it interferes with my ability to focus on tasks’; (4) Emotional inattention [EAD], four items about difficulties in recognizing and realizing one’s own emotions, e.g., ‘I am confused about how I feel’; and (5) Emotional confusion [EC], four items about difficulties in differentiating emotions while they are experienced, ‘I have difficulty distinguishing between different emotions’.

### 2.3. Procedure

This research is part of the internal project DIUA 302-2024 of the Universidad Autónoma de Chile, approved by the Ethics Committee, Act N° CEC 12-24. The study fully complied with the deontological guidelines for scientific research referred to in international agreements, specifically with what was stated in the Singapore declaration (Resnik and Shamoo 2011) and Helsinki declaration (World Medical Association 2013). Likewise, it adhered to the safeguarding of information in compliance with current regulations in Chile, as provided in Law 20,120 (MINSAL 2006). The above was explained to the participants through an informed consent and confidentiality notice. This questionnaire was sent to various schools in the country, asking the directors to disseminate it to their teachers. The questionnaire was administered online, where participants had the possibility of contacting the researchers if they had questions and voluntarily deciding whether to participate or not. The estimated time to answer the instrument was approximately 10 min. The survey was conducted online and remained open from 4 September to 6 October 2023, totaling 33 days. Due to the nature of the scales and the configuration of the web form, no missing data were recorded. Participants had to complete all items before submission, ensuring a complete dataset.

### 2.4. Analysis Plan

First, the normality of all items was evaluated using the Kolmogorov–Smirnov test, in order to determine the subsequent data analysis methods. Subsequently, a Confirmatory Factor Analysis (CFA) was applied to estimate the fit of the theoretical model to the data, using the method of maximum likelihood estimation with robust standard errors and a mean- and variance-adjusted test statistic (MLMV). Goodness-of-fit indices were calculated in four ways: (1) Chi-square statistic; (2) Root Mean Square Error of Approximation (RMSEA); (3) Comparative Goodness of Fit Index (CFI) and; (4) Tucker–Lewis index (TLI). Values less than 0.05 in RMSEA were considered excellent and for both CFI and TLI, values greater than 0.9 were considered good and values greater than 0.95 were considered excellent, as per Hu and Bentler (1999).

Convergent validity was evaluated by expecting each subscale to meet the following criteria: (1) indicators with standardized loadings must have values greater than 0.5 and a level of statistical significance with a *p*-value less than 0.05; (2) average variance extracted (AVE) values must be greater than 0.5; and (3) composite reliability must have values greater than 0.7 (Hair et al. 1999). Discriminant validity was evaluated using the comparison between the shared variance and the average variance extracted. If the AVE of a latent variable was greater than the square of the correlation between it and the other subscales, we considered that to be good evidence of discriminant validity (Fornell and Larcker 1981).

Composite reliability was used to evaluate the reliability of the instrument, through for the McDonald omega coefficient (McDonald 1999), for which values greater than 0.65 are admissible, values greater than 0.7 are acceptable, between 0.8 and 0.9 are good, and values equal to or greater than 0.9 are excellent (Ventura-León and Caycho-Rodríguez 2017).

In order to fulfill the central purpose of the study, a measurement invariance analysis was carried out by categories according to the groups of interest for the research (Gender, Macrozone, and Educational level), which allows us to determine to what degree the scale measures different groups in the same way. Establishing that an instrument complies with measurement invariance (metric or scalar), allows us to analyze the presence of statistically significant differences between groups in the variables measured by the scales. To test configural invariance, the goodness-of-fit indices referred to for a CFA were considered. Metric invariance is achieved if, with respect to the configural model, there is a delta of the RMSEA less than 0.015 and/or a delta of the CFI less than 0.01. The same values are considered between the scalar and metric model to establish scalar invariance. We took into account considerations by Chen (2007) that, for sample sizes with unequal groups less than 300, the variation in CFI must be equal to or less than 0.005 and the change in RMSEA equal to or less than 0.01.

Once the above steps were achieved, it was possible to examine whether there were differences between groups. In this regard, given that the distribution of the data does not resemble a normal distribution, the differences in the categories with more than two groups were reviewed through the Kruskal–Wallis test and the differences between pairs of groups were calculated using the Mann–Whitney U test. Subsequently, the median was compared and the differences reviewed, with statistical significance at the 0.05 level, in addition to analyzing the statistical power and effect size (Faul et al. 2009).

The influence of age on participants’ emotional regulation was also evaluated and the interaction between age and gender measured for each latent variable, employing Structural Equation Modeling (SEM) analysis. The goodness fit indexes criteria used for CFA were considered. Finally, cut-off points were calculated for the latent variables using K-Means cluster analysis.

SPSS v.27 (IBM Corp 2020) was used to perform normality tests, hypothesis testing, descriptive statistics, and K-Means cluster analysis. CFA, measurement invariance analysis, and SEM analysis were performed with RStudio (RStudio Team 2022). For AVE, reliability, convergent, and discriminant validity calculations, Excel v.16 (Microsoft 2023) was used. For the measurement of effect size and statistical power, G*Power software was used (Buchner et al. 2020).

## 3. Results

**Figure 1:**
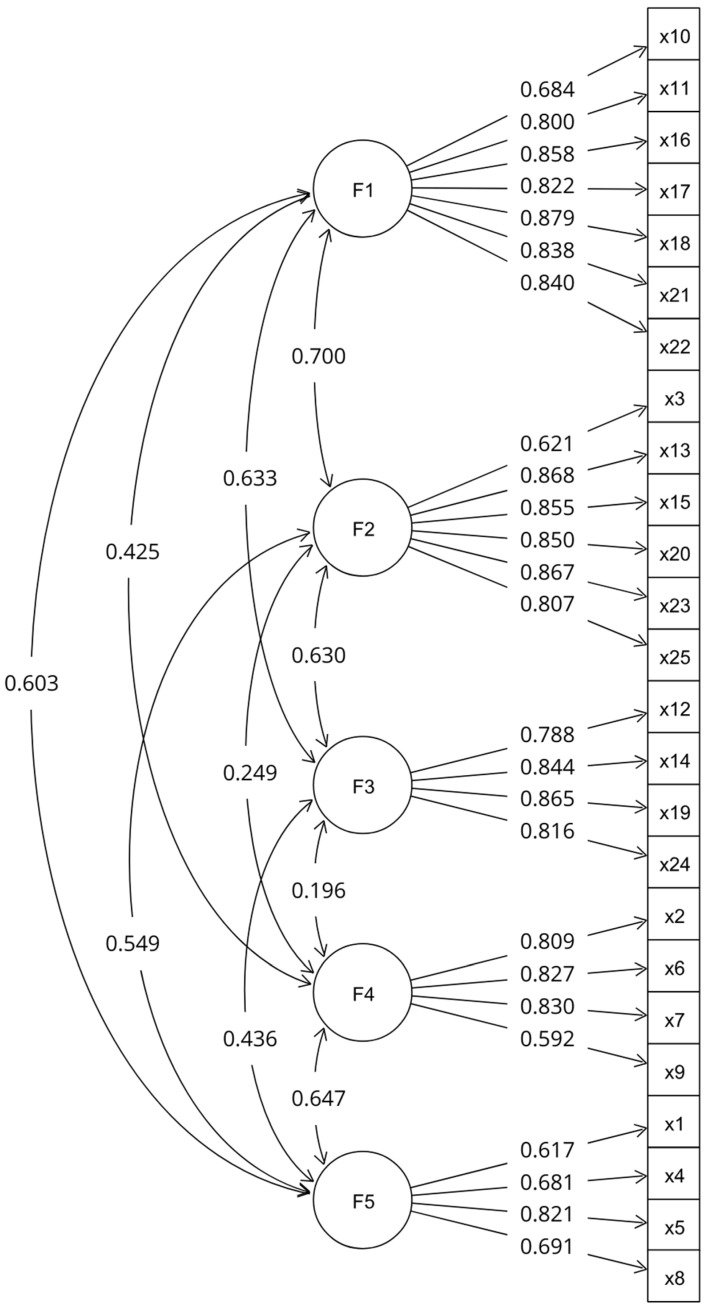
CFA path diagram. Source: prepared by the authors.

The Kolmogorov–Smirnov test shows that the data do not resemble a normal distribution (*p* < 0.001) in all the instrument indicators. The goodness of fit indices show a good fit of the proposed model (Figure 1) to the data (X2 = 819.925; DF = 265; ratio = 3; *p* < 0.001; RMSEA = 0.044; CFI = 0.948; TLI = 0.941).

Convergent validity is evident given that the standardized loadings of the indicators range from 0.592 to 0.879 with the statistical significance *p* < 0.001. The AVE of the subscales show values from 0.5 to 0.7 and the composite reliability for the latent variables shows values from 0.788 to 0.934. Then, the AVE of the subscales is greater than the square of the correlations between it and the other latent variables, which allows for the establishing of the discriminant validity between the dimensions. Furthermore, the instrument shows good reliability indicators, since each dimension presents ω values ≥ 0.8 (Table 2).

The CFA applied to all groups of the explored categories (Gender, Macrozone and Educational level), showed a good fit of the proposed model to the data (Table 3). Scalar invariance was achieved in all categories (ΔCFI < 0.01, ΔRMSEA < 0.015) (Table 4).

As shown in Table 5, men and women showed statistically significant differences for three components of emotion dysregulation. Women reported more negativity with regard to their own emotions than men did, and they also reported feeling more interference from these emotions in their everyday behaviors than men did. Women also reported being less clear about what they were feeling than men did.

As for age, the SEM analysis shows a good fit (RMSEA = 0.045; CFI = 0.947; TLI = 0.939). With the exception of the Emotional neglect factor, there were statistically significant negative relationships with emotional dysregulation for age, i.e., the older the age, the lower the emotional dysregulation (Table 6).

Regarding the interaction of age with gender, the SEM analysis fitted the data well (RMSEA = 0.040; CFI = 0.939; TLI = 0.935). Male teachers show statistically significant relationships in two factors, while female teachers show statistically significant relationships in four factors (Table 7).

**Figure 2:**
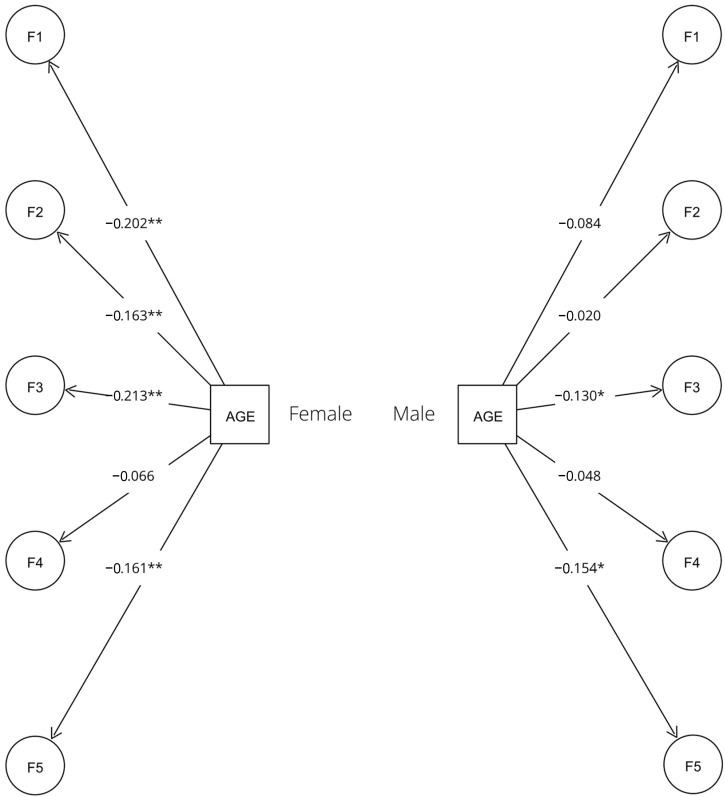
SEM female–male path diagram. Source: prepared by the authors. Notes: * indicates significance at the 0.05 level; ** indicates significance at the 0.01 level. For clarity, this figure has been simplified to illustrate the relationship between age and gender only.

The SEM path diagram of men and women in relation to age can be seen in Figure 2.

The K-means cluster analysis allowed grouping the results of each factor and of the total scale into low, medium and high levels. Considering the statistically significant differences found between men and women, separate cut-off scores were created according to gender (Table 8).

## 4. Discussion

The present study conducted a measurement invariance analysis of the Difficulties in Emotional Regulation Scale (DERS-E) in a sample of teachers from the Chilean school system, and then assessed whether there were significant differences in emotional dysregulation as a function of gender and age. The confirmation of scalar invariance implies that one can confidently compare the averages of the latent factors between groups, knowing that any differences are attributable to actual variations in the constructs measured and not to the properties of the scale itself (Cheung and Rensvold 2002; Steinmetz et al. 2009).

Statistically significant differences in emotional dysregulation were observed between men and women. Women showed higher levels of emotional rejection, daily interference, and less clarity in what they feel compared to men, which is consistent with previous studies suggesting that women tend to report greater difficulties in emotional regulation. In this regard, the work of Dzokoto et al. (2018) on cultural norms and emotional regulation highlights that cultural expectations can significantly influence how people manage and express their emotions. Women, in many cultural contexts, face higher expectations for emotional regulation and expression, which may result in greater worry about and greater pressure to control and express their emotions in a socially acceptable manner (Brody and Hall 2010). Further, women, due to cultural norms, are seen as more empathic and have a greater emotional burden to display empathic behaviors and manage emotions effectively (Bagrowska and Gawęda 2023). This cultural burden may lead to women having to work harder to meet these expectations, which may influence their emotional well-being and self-perception.

Affective empathy involves experiencing emotions in response to the emotional experiences of others, which can lead to personal distress when confronted with intense suffering, often resulting in avoidance behavior (Stevens and Taber 2021). In the context of this study, this could lead to an underestimation of the child’s situation or a tendency toward psychological distancing characterized by emotional detachment (Fabris et al. 2024; Stevens and Taber 2021; Pang et al. 2023).

We also found emotional dysregulation differences by age, with fewer difficulties in emotional regulation, particularly in the dimensions of emotional rejection, daily interference, and emotional confusion, for older teachers. We suspect that older teachers tend to develop better emotional regulation skills, possibly due to greater experience in managing emotions in educational contexts, which allows them to better cope with the emotional demands of their role (Isaacowitz and English 2024). Greater exposure to situations that require effective emotional regulation may contribute to the development of more adaptive strategies over time (Paredes-Rivera et al. 2021).

Better emotion regulation with age was most evident for female teachers, as demonstrated by the statistically significant interaction between age and gender. In men, age was associated with a decrease in the daily interference of emotions and emotional confusion, which may reflect a process of emotional adaptation related to accumulated experience and the internalization of more effective regulation strategies. In women, however, age was related to a reduction in four of the five dysregulation scales: emotional rejection, emotional dyscontrol, daily interference of emotion, and emotional confusion. This highlights how emotion regulation may evolve differently according to context and lived experiences (Fuentes-Vilugrón et al. 2022). These findings also highlight the potential for change when designing intervention programs that promote effective emotional regulation in the educational setting. Recent work in which younger, middle-aged, and older individuals reported their use of 17 emotion regulation tactics and the perceived success of these tactics suggested important age differences in both strategy, use, and success (DiGirolamo et al. 2023). For example, down-regulating negative affects was a strategy most often used by younger adults but it was less successful in achieving emotion regulation for them. It may be that older teachers have developed increased usage of positivity as a way of coping with the daily stressors in the classroom, and that female teachers are employing it most successfully. Future research would benefit from inclusion of both positive up-regulation and negative down-regulation strategies to assess their success for teachers by gender as well as age.

The capacity for emotional regulation in teachers is fundamental for their personal well-being, and may well stem from teachers’ own previous adverse childhood experiences; without resolving these dysregulatory experiences and developing resulting strategies, teachers may find themselves reliving and recreating these patterns in their own adult lives (Hubel et al. 2020). Importantly, dysregulated teachers are likely to have less capacity for developing an effective learning environment for students. Teachers who have dysregulated backgrounds may also detach themselves from the events in the classroom, and indeed from individual students as well in order to protect their emotional well-being. In so doing, they then become prone to less skillful classroom management, and reduced reporting of serious and identifiable problems for their students (e.g., child abuse situations (Fabris et al. 2024)). The incorporation of gender-tailored emotional regulation strategies can significantly improve teachers’ quality of life and, consequently, their professional performance and their ability to positively influence their students (Taxer and Gross 2018).

The cut-off points proposed in this study for the DERS-E are specific to teachers in the Chilean school system, and are relevant given the crucial role of teachers in the emotional socialization of students (Gross and John 2003; Gratz and Roemer 2004; Nolen-Hoeksema 2012; Dzokoto et al. 2018; Bagrowska and Gawęda 2023). These cut-off points allow for identifying levels of emotional dysregulation that require intervention and provide a basis for the implementation of emotional support programs. The specificity of these cutoff points differs slightly from previous studies in the Chilean general population (Guzmán-González et al. 2014), which highlights the importance of taking into account the particular characteristics of the teaching collective when designing assessment tools and emotional intervention programs (Gross and John 2003; Garnefski et al. 2001; Muñoz-Martínez et al. 2016).

The limitations of this study include a non-representative sample size and a heavy reliance on self-reported data, which may introduce social desirability bias and limit the generalizability of the findings. The gender imbalance (80.3% women and 19.7% men) mirrors the actual composition of the Chilean teaching workforce but might present challenges when extrapolating findings across genders. The age distribution in the sample suggests a cohort with an average age of 37 years, which may not capture the full range of emotional regulation skills across different life stages. The study also lacks data on the specific subjects taught, which could provide insights into the unique stressors linked to different teaching disciplines. Additionally, we did not compare the DERS-E with other tools that measure emotional regulation, as well as assessments of core personal resources and mental health (e.g., depression, anxiety disorders, and burnout). Because we began with a measure utilized for the general population, we also do not know if there are dysregulatory scales that are particularly relevant or unique for educators; these might need to be uncovered using qualitative research practices. We do think, however, that these might reveal the situations that most activate dysregulation for teachers rather than the actual emergence of different dysregulatory structures. Finally, the study does not consider how emotional dysregulation directly affects teachers’ daily practice, such as challenges in classroom management and the potential for burnout (Dekeyser et al. 2021; Doyle et al. 2024; Li 2023).

Future research should aim to explore the evolution of emotional regulation skills over time and in response to specific interventions. Incorporating complementary assessment methods, such as direct observations or third-party evaluations, would provide a more accurate picture of emotional regulation in educational contexts (Morris et al. 2017; Althubaiti 2016). Research should also investigate the inclusion of contextual and personal factors, such as the type of educational institution, socioeconomic environment, and emotional competency training, to gain a deeper understanding of the determinants of emotional regulation and its implications for teachers’ well-being and professional performance. Examining the effectiveness of emotional skills training programs for teachers could assess their impact on emotional dysregulation, students’ well-being, and academic performance (Extremera et al. 2019; Guzmán-González et al. 2014), as some research already shows the efficacy of DBT training in the acceptance and goals components (Huerta-Hernández et al. 2021). Moreover, research should consider how emotional dysregulation affects classroom management, teacher–student relationships, and the creation of structured learning environments. Developing and implementing emotional skills training programs that equip teachers with effective tools to manage emotions is vital for creating positive learning environments and enhancing teaching effectiveness (Brackett et al. 2010; Ornaghi et al. 2023; Jennings and Greenberg 2009; Wang et al. 2024).

## 5. Conclusions

The findings confirm the measurement invariance of the DERS-E across the Gender, Macrozone, and Educational levels, although differences were observed in the Mann–Whitney U-test. These results underscore the tool’s robustness in assessing emotional dysregulation among Chilean teachers while also highlighting areas where gender and age may influence emotional regulation strategies. Specifically, female teachers reported higher levels of emotional rejection, greater everyday interference of emotions, and less awareness of what they were feeling than male teachers, reflecting cultural norms and expectations around emotional expression. The use of SEM allowed for nuanced insights into how age impacts emotional regulation, revealing a general trend toward improved regulation skills with increasing age, but also a pattern such that female teachers seemed to benefit the most with age. These insights are important when designing interventions that address the specific needs of different demographic groups within the teaching profession. By acknowledging these differences, educational policymakers and practitioners can better support teachers’ emotional well-being and professional development, ultimately enhancing the quality of education (Isaacowitz and English 2024; Guzmán-González et al. 2020; Bollen and Curran 2006; Little 2013).

In conclusion, this study reveals the importance of considering gender and age factors in teachers’ emotional regulation, as evidenced by the fulfillment of all hypotheses. The results provide a solid basis for developing interventions that promote emotional well-being in educational contexts, which is fundamental for educational success and the comprehensive development of students. Implementing emotional regulation strategies tailored to teachers’ specific needs can significantly improve the quality of education and the learning environment, benefiting both teachers and students. Emotional regulation is a crucial skill that significantly impacts teachers’ well-being and professional performance. Therefore, it is essential to develop and support emotional skills training programs that can help teachers better manage their emotions and create a positive and effective learning environment.

## Figures and Tables

**Table 1:** Instruments to evaluate ER.

Instrument Name
The Meta-emotion Interview-Parent Version (meI-pv), (Katz and Gottman 1999; Shipman et al. 2007)
Cognitive Emotion Regulation Questionnaire (ceRQ) (Garnefski et al. 2001)
Emotion Regulation Questionnaire (ERQ) (Gross and John 2003)
Emotional Regulation: Implicit Assessment Test (ER-IAT) (Greenwald et al. 2003)
Inventory of Cognitive Affect Regulation Strategies (IcaRUS) (Kamholz et al. 2006)
Emotional Approach Coping (Stanton et al. 2000)
Emotional Regulation During Test-Taking (ERT) (Schutz et al. 2004)
Levels of Emotional Awareness Scale (leaS) (Lane et al. 1990)
Emotional Instability Scale (Ine) (Caprara and Pastorelli 1993)
Difficulties in Emotion Regulation Scale (DeRS) (Gratz and Roemer 2004)
Anger Control Inventory (Hoshmand and Austin 1987)
Measurement of Affect Regulation Styles (MARS) (Lassen and Prizmic 2018)
Measurement of Affect Regulation Styles (MARS Expanded) (Rovira et al. 2012)

Source: prepared by authors.

**Table 2:** Reliability; convergent and discriminant validity.

	Omega	AVE	F1	F2	F3	F4	F5
F1	0.9	0.671		0.490	0.401	0.181	0.301
F2	0.9	0.666	0.700		0.397	0.062	0.301
F3	0.9	0.687	0.633	0.630		0.038	0.190
F4	0.9	0.594	0.425	0.249	0.196		0.419
F5	0.8	0.499	0.549	0.549	0.436	0.647	

Source: prepared by the authors. Note: below the diagonal are the correlations between factors. Above the diagonal are the correlations between factors squared.

**Table 3:** CFA groups.

Categories	Group	N	X2	DF	RMSEA	CFI	TLI
Gender	Female	850	789.005	265	0.048	0.946	0.939
	Male	209	360.244	265	0.041	0.923	0.909
Macrozone	North	132	337.264	265	0.045	0.933	0.924
	Center	332	444.987	265	0.045	0.935	0.927
	South	222	375.109	265	0.043	0.943	0.936
	Metropolitan	373	455.807	265	0.044	0.941	0.933
School level	Primary	643	616.151	265	0.045	0.945	0.938
	Secondary	416	436.571	265	0.039	0.953	0.947

Source: prepared by the authors. Note: in all groups, the Chi-square statistic showed a *p*-value less than 0.001.

**Table 4:** Contrast in measurement invariance of categories.

Category	Level	RMSEA	CFI	ΔRMSEA	ΔCFI
Gender	Configural	0.042	0.941	-	-
	Metrics	0.041	0.942	0.001	0.001
	Scalar	0.041	0.941	0.000	0.001
Macrozone	Configural	0.044	0.938	-	-
	Metrics	0.043	0.937	0.001	0.001
	Scalar	0.042	0.936	0.001	0.001
School level	Configural	0.042	0.949	-	-
	Metrics	0.041	0.949	0.001	0.000
	Scalar	0.041	0.949	0.000	0.000

Source: prepared by the authors.

**Table 5:** Differences in perception by gender. Mann–Whitney U test.

	Female	Male					
	n = 850	n = 209					
	Average	Average					
Factors	Range	Range	Z	U	*p*	1 − β	*d*
Emotional rejection	548.73	453.82	−4.023	72,903.0	<0.001	0.81	0.33
Lack of control	538.35	496.05	−1.796	81,730.0	0.072	0.70	0.18
Everyday interference	546.65	462.30	−3.579	74,675.0	<0.001	0.59	0.28
Emotional neglect	539.91	489.72	−2.132	80,405.5	0.033	0.39	0.15
Emotional confusion	537.80	498.28	−1.681	82,196.0	0.093	0.37	0.11

Source: prepared by the authors.

**Table 6:** Relationship of age with emotional dysregulation.

Factors	γ	*p*	Range	L	U
Emotional rejection	−0.169	<0.001	−0.169	−0.240	−0.097
Lack of control	−0.123	<0.001	−0.123	−0.193	−0.053
Everyday interference	−0.186	<0.001	−0.186	−0.261	−0.112
Emotional neglect	−0.057	0.069	−0.057	−0.139	−0.024
Emotional confusion	−0.181	<0.001	−0.181	−0.263	−0.099

Source: prepared by the authors.

**Table 7:** Relationship of age and gender with emotional dysregulation.

	Female				Male			
Factors	γ	*p*	L	U	γ	*P*	L	U
Emotional rejection	−0.202	<0.001	−0.281	−0.122	−0.084	0.124	−0.226	0.057
Lack of control	−0.163	<0.001	−0.239	−0.087	0.020	0.735	−0.135	0.176
Everyday interference	−0.213	<0.001	−0.296	−0.131	−0.130	0.021	−0.275	0.015
Emotional neglect	−0.066	<0.065	−0.159	0.026	−0.048	0.418	−0.199	0.104
Emotional confusion	−0.191	<0.001	−0.281	−0.101	−0.154	0.030	−0.337	0.029

Source: prepared by the authors.

**Table 8:** Cut-off scores.

	Low		Medium		High	
Scale	Male	Female	Male	Female	Male	Female
Emotional rejection	10	11	20	21	30	32
Lack of control	8	8	15	16	22	24
Everyday interference	7	7	12	13	17	18
Emotional neglect	8	8	13	13	18	18
Emotional confusion	8	8	11	11	15	15
DERS-E	52	54	71	73	90	96

Source: prepared by the authors.

## Data Availability

The original contributions presented in the study are included in the article, further inquiries can be directed to the corresponding authors.
